# The Gap Between AI and Bedside: Participatory Workshop on the Barriers to the Integration, Translation, and Adoption of Digital Health Care and AI Startup Technology Into Clinical Practice

**DOI:** 10.2196/32962

**Published:** 2023-05-02

**Authors:** Iredia M Olaye, Azizi A Seixas

**Affiliations:** 1 Department of Medicine Weill Cornell Medicine Cornell University New York, NY United States; 2 Covered By Group Covered By Health Newark, NJ United States; 3 Media and Innovation Lab Department of Informatics and Health Data Science The University of Miami Miller School of Medicine Miami, FL United States

**Keywords:** digital health, startups, venture capital, artificial intelligence, AI translation, clinical practice, early-stage, funding, bedside, machine learning, technology, tech, qualitative, workshop, entrepreneurs

## Abstract

**Background:**

Artificial intelligence (AI) and digital health technological innovations from startup companies used in clinical practice can yield better health outcomes, reduce health care costs, and improve patients' experience. However, the integration, translation, and adoption of these technologies into clinical practice are plagued with many challenges and are lagging. Furthermore, explanations of the impediments to clinical translation are largely unknown and have not been systematically studied from the perspective of AI and digital health care startup founders and executives.

**Objective:**

The aim of this paper is to describe the barriers to integrating early-stage technologies in clinical practice and health care systems from the perspectives of digital health and health care AI founders and executives.

**Methods:**

A stakeholder focus group workshop was conducted with a sample of 10 early-stage digital health and health care AI founders and executives. Digital health, health care AI, digital health–focused venture capitalists, and physician executives were represented. Using an inductive thematic analysis approach, transcripts were organized, queried, and analyzed for thematic convergence.

**Results:**

We identified the following four categories of barriers in the integration of early-stage digital health innovations into clinical practice and health care systems: (1) lack of knowledge of health system technology procurement protocols and best practices, (2) demanding regulatory and validation requirements, (3) challenges within the health system technology procurement process, and (4) disadvantages of early-stage digital health companies compared to large technology conglomerates. Recommendations from the study participants were also synthesized to create a road map to mitigate the barriers to integrating early-stage or novel digital health technologies in clinical practice.

**Conclusions:**

Early-stage digital health and health care AI entrepreneurs identified numerous barriers to integrating digital health solutions into clinical practice. Mitigation initiatives should create opportunities for early-stage digital health technology companies and health care providers to interact, develop relationships, and use evidence-based research and best practices during health care technology procurement and evaluation processes.

## Introduction

### Background

Emergent technologies in health care, such as digital health care technologies and artificial intelligence, have changed and shaped clinical care for patients by filling gaps in the current health care delivery system. One primary driver for the digitization of health care is early-stage digital health care startups [1]. Early-stage digital health companies are typically new or emerging startup business ventures that solve small and overlooked problems in the health care ecosystem or disrupt the health care market with innovative solutions and reach underserved markets [2]. Some of the most innovative technologies emerge from early-stage digital health technology and AI startup companies [3]. For example, the National Basketball Association, the second largest professional sports league in the United States, used Oura Health, which at the time was a company with Series A financing. Specifically, they used Oura Health's “Oura Ring,” a health-tracking device that provides interesting data, particularly for monitoring physiological information and early COVID-19 illness detection monitoring services during the 2020 season [4,5]. This technology was critical for maintaining player safety and keeping the National Basketball Association season going during the COVID-19 pandemic, preserving roughly US $10 billion in revenue [6].

**Figure 1:**
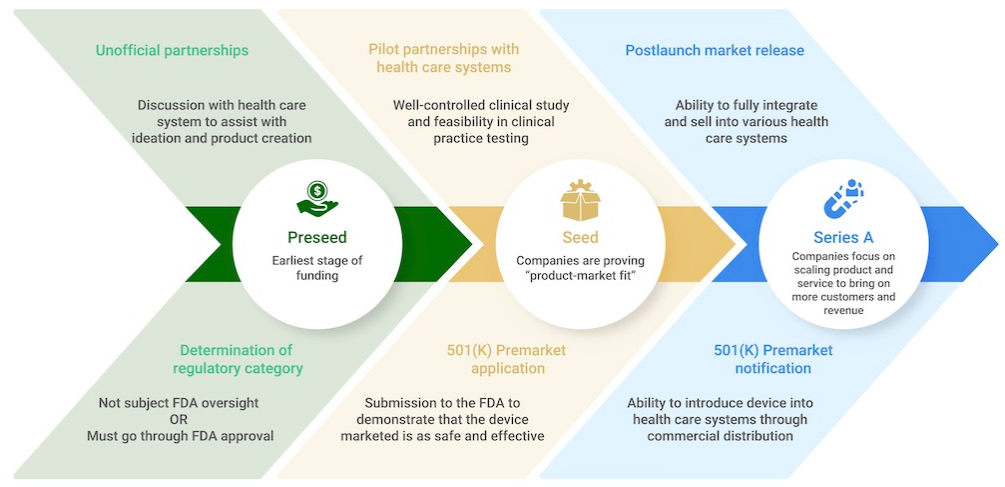
Progression of early-stage digital health and health care artificial intelligence startup funding, regulation, and integration into clinical practice. FDA: Food and Drug Administration.

Early-stage digital health companies, often referred to as “startups,” which produce advanced technological solutions such as artificial intelligence (AI), machine learning, and digital health interventions, pass through different critical development stages [7]. Currently, the essential steps in the transition from a nascent startup to an organization capable of sustained and profitable growth are not readily apparent [8]. In Figure 1, the stages are identified for a typical early-stage digital health company. The stages that are displayed correspond to the regulatory and health care system integration that is appropriate to them and are frequently overlapping. A digital health company's ability to lay the foundation for product development, regulation, and health care system partnerships and integration during the early-stage period dramatically influences the company's success.

Despite the importance of health care systems' relationships to early-stage digital health companies, partnerships integrating machine learning, AI or digital health care technologies into clinical practice are plagued with many challenges and thus are generally slow, preventing implementation in places where it could be most beneficial [9]. For example, health care technology procurement—defined as the process by which health care systems and organizations evaluate and purchase innovative digital health technologies, goods, devices, or services from external companies—can delay the timely integration and rapid adoption of technology in clinical practice [10]. Moreover, clinicians, patients, payers, and regulators often mention that inconsistent data on technology effectiveness, limited evidence on clinical validation, and the impact of digital health on the overall health ecosystem are the main reasons behind the slow adoption and integration of these technologies in clinical practice [11]. However, explanations for the slow and limited adoption and integration of digital technologies have been largely speculative and, to our knowledge, have not been systematically studied and are thus largely unknown. This is especially true from the vantage point of early-stage digital health companies, which face unique challenges in getting their products into health systems, compared to larger health care technology companies and, thus, are unable to sell directly to health organizations [12].

### Objectives

The aim of the study was to describe the barriers to integrating early-stage digital health technologies in clinical practice and to suggest intervention and education strategies to mitigate the barriers. This is a pressing issue, as many patients cannot wait long periods for the necessary technologies to be adopted in their city or town. Although many early-stage digital health technology companies bring exciting innovations to clinical care, there are significant barriers to integrating these technologies into clinical practice and health care systems [13-17]. In addition, the needs and perspectives of early-stage digital health and health care AI entrepreneur stakeholders are not always considered in health care technology research, resulting in the lack of information available for both early-stage digital health technology companies and health care institutions [2].

Previous reviews and studies highlighted the limitations in the quality of literature on the consumer aspect of digital health technologies, insights into entrepreneurial orientation and motivation, and the perspectives of founders or entrepreneurs [2,11,18]. The aim of the study was to describe the barriers to integrating early-stage digital health and AI technologies in clinical practice and health care systems by engaging stakeholders in current companies going through these critical development stages. As stakeholder engagement can improve research quality and relevance, we felt they were the ideal target sample for our first foray into this line of research [19].

## Methods

### Study Design

An exploratory, descriptive, qualitative study was conducted using a stakeholder focus group workshop design on early-stage digital health and health care AI entrepreneurs who are leaders of companies that develop digital health technologies [20]. A stakeholder focus group workshop was chosen as an appropriate data-collection tool because focus groups bring study participants to discuss a topic on facilitating trends and identification of critical problems, solutions, and experiences, allowing their specific perspectives and insights to emerge [21].

Thus, a stakeholder focus group workshop is a practical way to collect information from stakeholders, encouraging group interactions. This design provides in-depth insight into under-researched areas by interviewing stakeholders and experts to give an in-depth description.

We conducted a stakeholder focus group workshop facilitated by the principal investigator and two trained assistants, which lasted approximately 65 minutes [22]. All discussions were audio recorded. The focus group consisted of early-stage digital health and health care AI entrepreneurs to understand their perspectives on the integration of their technology solutions into clinical practice and to assess the barriers and facilitators in the integration of early-stage digital health innovations into clinical practice. We synthesized the participants' recommendations from these learnings to create a road map to mitigate the barriers to integrating early-stage or novel digital health technologies in clinical practice.

### Recruitment and Screening

The participants were recruited for the workshops using snowball and convenience sampling [19]. First, they were invited from a convenience sample of entrepreneurs in the population available to the researcher. They were then asked to identify other members of their network who might be interested in participating in the study. Sampling continued until data saturation, which occurred with a sample size of 10 individuals [23]. To ensure that all participants had the same background, we focused on digital health technologies for cardiovascular medicine [24]. Inclusion criteria for this research study were as follows: (1) English speaking; (2) 18 years of age or older; (3) founder, chief executive officer, or digital health entrepreneur; and 4) leader of a digital health solution for cardiovascular medicine.

### Workshop Procedure

Participants who confirmed participation in the workshop had to sign an informed consent form. At the start of the workshop, the principal investigator gave an evidence presentation to ensure that the stakeholders were familiarized with consistent information about the workshop. Evidence presentations are used in expert elicitations to capture and present all pertinent information that stakeholders rely on to formulate their opinion [25]. The evidence presentation included a brief introduction to the area of inquiry for the evaluation [26].

We aimed to optimize internal validity by providing access to the current data so the stakeholder could form opinions based on their different expertise and experiences. Optimizing internal validity was necessary to maintain the rigor of qualitative research and ensure the research results were trustworthy and credible. The presentation offered basic definitions of health care innovation integration, product definitions, summarized statistics, and provided an overview of the current state of the early-stage innovations integration into clinical practice [27-29]. The workshop was approximately 65 minutes and was facilitated by the principal investigator. The stakeholders were asked to discuss barriers related to the rapid integration of their early-stage digital health solutions into clinical health care systems and operations. To account for social desirability bias, defined as “the tendency of research subjects to choose responses they believe are more socially desirable or acceptable rather than choosing responses that are reflective of their true thoughts or feelings,” the participants shared their opinions privately by writing in a notebook in addition to verbally in the group discussion [29,30].

### Data Collection and Analysis

Data collection consisted of the participants listing barriers to integrating their products into clinical practice in a written notebook and the audio recording of the discussion. Using Braun and Clarke's inductive thematic analysis approach, we analyzed the transcripts and used NVivo 12 (QSR International) to organize, query, and explore data for thematic convergence [19]. The research team first transcribed the audio recordings verbatim. After transcription, the study team, consisting of 3 individuals, independently reread the transcripts with the audio recording to verify transcription accuracy and familiarize themselves with the qualitative data. In cases where there was disagreement, the study team listened to the audio recording together to finalize the accuracy of the transcribed content. Once transcription was complete, the study team generated four overarching themes of barriers based on repetition and patterns producing the results. Data were analyzed until we reached data saturation [23].

### Ethical Considerations

The stakeholders were informed orally during the workshop and in writing about the study's objective, privacy considerations, and voluntary participation. The participants' notebooks and audio and transcribed discussions were kept private, and their documents remained anonymous. The research group collected written informed consent.

## Results

### Participant Characteristics

To provide insight into the unique challenges faced by early-stage digital health and health care AI entrepreneurs, we conducted a multidisciplinary stakeholder workshop with 10 participants (n=4, 40% female and n=6, 60% male participants) representing the following groups of stakeholders: digital health entrepreneurs (4/10, 40%), health care AI entrepreneurs (4/10, 40%), a digital health entrepreneur turned venture capitalist (1/10, 10%), and a physician and digital health entrepreneur (1/10, 10%). Table 1 describes the participants in the workshop focus group.

**Table 1:** Description of stakeholder participants in the workshop focus group (n=10).

Stakeholder	Description of participants	Value, n (%)
Digital health entrepreneurs	Founder or chief executive officer of a preprofit company with a digital health solution for cardiovascular medicine	4 (40)
Health care AI^a^ entrepreneurs	Founder or chief executive officer of the preprofit company with an AI health solution for cardiovascular medicine	4 (40)
Digital health entrepreneur turned venture capitalist	Previous founder of preprofit company with a digital health solution for cardiovascular medicine turned venture capitalist (funder)	1 (10)
Physician and digital health entrepreneur	Physician and current founder of a company with an AI health solution for cardiovascular medicine	1 (10)

^a^AI: artificial intelligence.

### Themes and Barriers to Integrating Early-Stage or Novel Digital Health Technologies in Clinical Practice and Health Care Systems

Based on the written list in the notebooks and the transcribed discussion, we identified the following four overarching categories, as well as numerous sub-barriers, in the integration of early-stage or novel digital health technologies in clinical practice and health care systems: (1) lack of knowledge on health care system technology procurement protocols and best practices, (2) demanding regulatory and validation requirements, (3) challenges within the health care system technology procurement process, and (4) disadvantages of early-stage digital health companies compared to large technology conglomerates, as displayed in Table 2. Below, we provide a summary of each category of barriers, reported thematically in four overarching categories, followed by exemplifying quotes.

**Table 2:** List of barriers of digital health and health care AI^a^ in integration of their innovations into clinical health care systems and operation from the workshop.

Themes	Barriers
Knowledge on health care systems' technology procurement process	Lack of knowledge on health care systems' technology procurement protocols Limited access to best practices and strategies for successful technology procurement Venture funding leads more companies to sale directly to employers Lack of awareness on how to reach and educate providers on product offerings
Digital health innovations from large technology companies	Competing with large technology companies Lack of large marketing departments Lack of broad network of connections in comparison to larger companies Lack of networking and financial resources in comparison to larger companies
Demanding regulatory and validation requirements	Strenuous regulatory, validation, and technology evaluation evidence required from health care systems Lack of funding for randomized controlled trials Inappropriate existing study design to evaluate digital health innovations Inability to publish study results in academic journals and other peer review mediums due to proprietary concerns Lack of ability to explain AI algorithms
Success in health care systems' technology procurement	Limited information and uniformity on the health care procurement process Lengthy sales cycle Strenuous marketing and networking process Lack of transparency on who the decision maker is Lack of funding to attend conference trade shows Limited resources to support a health care pilot that demonstrates financial and clinical ROI^b^

^a^AI: artificial intelligence.

^b^ROI: return on investment.

### Lack of Knowledge on Health Care Systems' Technology Procurement Protocols and Best Practices

The stakeholders expressed deficiencies in their knowledge of the health care sales cycle and implementation process of digital technologies into clinical practice. All members of the group mentioned there is limited information and uniformity on the health care procurement process. They argued that each client and clinical health care organization, system, or physician has a unique process for vendor purchasing and selection. Furthermore, they stated the knowledge on how to sell into a particular health organization is derived from best practices of colleagues and members of their professional network. Overall, all members of the group highlighted the difficulty of selling health innovations due to the lack of knowledge on the process.

It is an extremely complicated and frustrating process. Each client and clinical healthcare organization, system and/or physician has a unique process for vendor purchasing and selection. We have to learn them all.

Participant B; digital health entrepreneur

### Demanding Regulatory and Validation Requirements

The participants raised concerns about the strenuous regulatory, validation, and technology evaluation evidence that is required for their products to be used in clinical settings. Randomized controlled trials are the golden standard and oftentimes are the inappropriate study design for the evaluation of digital health innovations. When asked about their preferred methodology and validation processes and procedures for evaluating their technologies, the participants' answers varied. All of them shared and expressed confusion and frustration toward the challenges in the evaluation of digital health solutions. Only 2 participants stated they published study results in academic journals and other peer-reviewed mediums. The participants with AI solutions shared that potential clients require extensive detailed information on the back end of how their technology functions, especially products that have AI-driven decision-making capabilities. Explainability versus accuracy is a debate the entrepreneurs have with their teams constantly. To summarize, they are not sure if health care systems would prefer simpler AI innovations that are less accurate or complex AI innovations with high accuracy and low explainability.

Early-stage startups are severely disadvantaged. We have research that supports our product's usability, effectiveness, and safety, but it seems that everyone wants RCTs (randomized controlled trials). RCTs are extremely expensive. It is like a cost-benefit. If the benefit cannot keep up with the cost, our products will not be implemented into the practice.

Participant A; digital health entrepreneur

I've chaired medical device committees in various healthcare entities for many years. It sadly depends on the system. My current hospital prefers results from randomized control trials. My previous hospital relied on patient/provider testimonies and user research feedback to evaluate if we should buy a product. Large tech companies have 200 people in their sales, research and product development teams, who can find this info out.

Participant P; physician and digital health entrepreneur

### Challenges Within the Health System Technology Procurement Process

The participants were asked to name the top 3 health care system technology procurement barriers experienced by early-stage health care technology entrepreneurs. The overall response to this question was remarkable. Six (60%) participants commented on the length of the sales cycle. The average length of the sale cycle in the group was 13 months. A small minority of the participants indicated the marketing and networking process as one of the biggest hurdles. Surprisingly, all participants mentioned that the top barrier was lack of information on the appropriate decision maker and process. In the group, the most used strategy to connect with decision makers at hospital and health care systems was to cold-call each department and ask for a referral and contact information of specific personnel.

It's hard to find the right person to talk to. We have limited resources and, frankly, time. It is important to speak directly with a decisionmaker. The problem, though, is without a connection from my network, it is tough to reach out to them. The decision-maker varies depending on the organization.

Participant D; health AI entrepreneur

### Disadvantages of Early-Stage Digital Health Companies Compared to Large Technology Conglomerates

All participants were confident in large health care technology companies' role in the unique challenges of integrating their products into clinical practice. The participants identified numerous barriers, listed in Table 1, most notably, health care organization preference for large technology companies and uneven competition as well as funding barriers. Large technology companies and conglomerates have comprehensive marketing departments and more capabilities to hire the best health care enterprise sales talent in comparison to smaller companies.

Because of the unique nature of healthcare sales cycles, [digital health entrepreneurs] are recommended to raise more funding dollars at the early stage than other venture-backed technology companies. Basically, successfully integrating new technologies relies on the early-stage startup's availability to segment sufficient marketing dollars for the entire length of the sale process.

Participant E; digital health entrepreneur turned venture capitalist

## Discussion

### Principal Findings

The results of our study highlighted the barriers facing the integration of early-stage digital health innovations into clinical practice and health care systems. We identified 4 areas of barriers, as follows: lack of knowledge on health care systems' technology procurement protocols and best practices, demanding regulatory and validation requirements, challenges within the health care systems' technology procurement process, and disadvantages of early-stage digital health companies compared to large technology conglomerates.

### Lack of Digital Health Validation and Evidence

It remains an industry-wide challenge to evaluate digital health solutions and provide credible evidence, hindering adoption and widespread use in health care [12]. There needs to be more widely available clinical efficacy data on digital health care products available to clinical providers. Existing methodologies used in standard regulatory bodies and health care technology assessment standards are not equipped to evaluate sophisticated AI health care solutions [31,32]. In addition, there is not a uniform assessment of health care technology products with AI capabilities [33]. Boni [34] recommended emerging digital health organizations to formulate integrated multidisciplinary commercialization teams responsible for addressing the multidimensional value proposition across the company's life cycle. This team would focus on external reporting to regulatory bodies and clinical providers, which need information on the company's technology, business, marketing, reimbursement, and product offerings. Previous studies have shown that continuous advances in digital health, especially products with AI capabilities, can increase care efficiency and decrease operative time. However, complex AI algorithms and innovations sacrifice transparency and interpretability for prediction accuracy, and little is known about the process of value cocreation enabled by health care AI. Baxter et al [35] concluded that the successful implementation of algorithms in clinical practice requires algorithm predictions to create a large impact on patient care and provide results that clinicians can interpret quickly and correctly [35]. We found similar findings in the health care systems' procurement category. Our study participants clarified that demonstrating financial and clinical feasibility in a manner that is easily interpretable by clinicians was their top priority. Further research is needed to create best practices for AI-related health care technology assessment and integration into clinical practice.

### Early-Stage Digital Health Startup Financing

Digital health innovations and artificial intelligence in health care are financed by venture capital and debt. Venture capital firms and startup friendly banks provide the majority of the financing for early-stage startup companies [36,37]. Continued funding for the lifecycle of the company is contingent on performance and follow on investment. Thus, capital investment decisions affect the companies' innovation plans, market definition, and, ultimately, products and overall existence of the firm [38]. This was demonstrated recently in the crisis facing Silicon Valley Bank (SVB) Financial Group, which provided venture debt and other types of financing to early-stage companies. Venture capital firms shaped the future of digital health care innovation through the investment choices of the types of early-stage companies selected to receive funding. The year 2020 had the highest amount of venture funding in health care reported, with over US $14 billion invested across 440 US digital health companies [39]. There were also numerous initial public offerings and mergers and acquisitions, with digital cardiovascular health having the most activity.

All the early-stage health care technology entrepreneurs in the workshop alluded to their current funding status or aspiration of funding as the key driver to their choice to integrate their products into health care systems. Helminen et al [38] analyzed a database of health care AI companies from open web-based sources to determine the factors that correlate with the amount of venture capital funding raised from various AI health care startups. Digital health innovations that employed AI solutions in clinical practice received a decrease in funding compared to other consumer-facing companies. Their results were consistent with this study's findings, suggesting there is a significant connection between funding and choice of business model and how digital health companies decide to acquire customers.

### Competitive Landscape: Early-Stage Digital Health Versus Technology Conglomerates

Early-stage companies need more financial resources for robust health care enterprise sales and marketing departments [40]. Study participants listed the lack of mutual social connections, networking opportunities, as well as personnel and financial resources for preprocurement engagement as the top disadvantages early-stage companies must combat in comparison to their larger competitors. Oftentimes, early-stage health care technology companies may have superior technology offerings compared to the larger companies [40]. Larger companies have a fixed network of medical professionals and marketing teams made up of many employees. These companies can break through the market and sell their new technologies into clinical practice with ease.

Due to the unique nature of health care sales cycles, digital health entrepreneurs are recommended to raise more funding dollars than other venture-backed technology companies. In other words, successfully integrating new technologies into clinical practice relies on the early-stage startup's availability to segment sufficient marketing dollars for the entire length of the sale process [38]. Many digital health entrepreneurs are shifting to direct-to-consumer and direct-to-employer payment models instead of setting up direct revenue models with health care systems due to the barriers addressed above [41]. Early-stage digital health companies produce innovations crucial to clinical patients and workflows, but unless they are assisted to mitigate the barriers to health care system integration, they will choose to focus on direct-to-patient care models. Investment trends can therefore be concerning for future clinical practice digital health innovations [42].

### Recommendations to Mitigate Barriers to the Rapid Integration of Early-Stage Digital Health and AI Technologies Into Clinical Practice

To mitigate the barriers early-stage digital health and health care AI entrepreneurs experience when integrating technologies, we provide the following road map and recommendations (Table 3). First, provide continuing education opportunities on the health care technology procurement process for digital health entrepreneurs and create continuing education opportunities for health care providers and systems on the innovations from early digital health companies. Health providers and systems do not always have access to the most up-to-date and informative real-world evidence and market research, making it difficult for them to be aware of the newest health care technologies on the market [11]. This problem only escalates because digital health entrepreneurs are not aware of the desired validation studies needed for health care systems to evaluate their products. This lack of consistent data from digital health efficacy studies and research leads to problems in the health care technology procurement process. To combat this, we recommend educating the providers and early-stage health care technology companies on the process of health technology validation by allowing transparent, accurate, and readily available research. Specifically, we aim to assist parties or committees of health care institutions in better educating both the providers and earthy-stage tech companies to help bridge the very problematic communication gap between the two.

**Table 3:** Recommendations and road map to mitigate the barriers to integrating early-stage or novel digital health technologies in clinical practice.

Themes and barriers			Recommendations
**Knowledge on health care systems' technology procurement**			
	Lack of knowledge on health care system technology procurement protocols and best practices	Continuing education for digital health entrepreneurs, health care providers, and systems on innovations and the health care technology procurement process	
**Large technology companies' digital health innovations**			
	Disadvantages of early-stage digital health companies compared to large technology conglomerates	Special activation and initiatives to support early-stage startup health care procurement processes	
**Demanding regulatory and validation requirements**			
	Demanding regulatory and validation requirements	Improving research and best practices on integrating digital health technologies into clinical practice	
**Health care systems' technology procurement barriers by early-stage health care technology entrepreneurs**			
	Challenges within the health care system technology procurement process	Creation of opportunities for early digital health technology companies, venture capitalists, as well as health care providers and systems to interact and develop relationships	
	Lack of bandwidth at health care systems to properly evaluate digital innovations	Creation of new health technology departments in medical systems with the following roles: Chief Information Officer Chief Research Information Officer Chief Clinical Information Officer	

Second is the creation of opportunities for early digital health technology companies, venture capitalists, health care providers, health care systems, regulatory boards, and insurance companies to interact and develop relationships. As more early-stage companies decide to raise external funding, and as the number and size of digital health deals increase yearly, it is possible that uneven funding and regulatory patterns can alter the development of digital innovations for clinical use [42]. Those who fund, regulate, and purchase digital and AI health care innovations strongly influence which types of products and algorithms will be used in clinical practice [43].

It is clear from our investigation that there is a critical need for investors, payers, health care organizations, regulatory boards, and technology stakeholders to collaborate and strategize on the best processes to improve digital health care technology integration into clinical practice. An example of this type of collaboration can be found in Germany through the Digital Health Applications (DiGA) Act. Insured patients are entitled to be provided with DiGA, which can be prescribed by doctors and psychotherapists and are reimbursed by the health insurance fund [44]. In addition, digital health companies around the world can apply online for a fast-track listing of their DiGA at the Federal Institute for Drugs and Medical Devices in Germany and will receive 12 months funding with full access and reimbursement.

### Limitations

While our study provided valuable insight into early-stage technology integration in clinical practice, this study has several limitations. The small sample size in comparison to other study designs such as surveys might prevent generalizability of the study results into other contexts. Furthermore, the sampling method of targeting leaders of preprofit companies with a digital health solution for cardiovascular medicine may offer limited generalizability to the entire AI and digital health care technology community. Additionally, it is possible that some early-stage digital health and the health care AI entrepreneur groups may have created an atmosphere where participants did not feel comfortable expressing proprietary information such as sales strategies focusing on the barriers. Different entrepreneur stakeholders might have produced the same or different themes, although thematic saturation was noted and reached [23].

### Conclusions

We found that the barriers to implementing health care technologies into clinical practice are vast. Based on the narratives of early-stage digital health and health care AI entrepreneur stakeholders, it is apparent that these barriers prevent patients and providers from having access to the newest technologies in clinical practice. Health care and clinical care structures are failing to catch up with the rapid progress of the health care AI and digital medicine technology industry [45]. With the rise of new digital health and AI technologies such as generative AI, ChatGPT, and wearable health care technologies, there needs to be a comprehensive and cohesive framework to evaluate their safety and effectiveness and integrate them into clinical care. The barriers that early-stage health care technology entrepreneurs face must be mitigated for these innovations to have their true impact so that they improve clinical care delivery and patient outcomes. This research uncovered a range of problems in the rapid integration of emerging digital health and AI innovations into clinical care. However, there is not enough supporting research in this arena. Future research should explore best practices and strategies for successful digital health and AI technology integration into clinical care, focusing on their impact on patient outcomes and cost reduction.

## Abbreviations

AI: artificial intelligence
DiGA: Digital Health Applications
